# The Significance of *Exo1* K589E Polymorphism on Cancer Susceptibility: Evidence Based on a Meta-Analysis

**DOI:** 10.1371/journal.pone.0096764

**Published:** 2014-05-08

**Authors:** Fujiao Duan, Chunhua Song, Liping Dai, Shuli Cui, Xiaoqin Zhang, Xia Zhao

**Affiliations:** 1 Department of Hospital Infection Management, Affiliated Tumor Hospital of Zhengzhou University, Henan Tumor Hospital, Zhengzhou, Henan, China; 2 Department of Epidemiology, College of Public Health, Zhengzhou University, Zhengzhou, Henan, China; 3 Henan Key Laboratory of Tumor Epidemiology, Zhengzhou, Henan, China; 4 College of Professional Study, Northeastern University, Boston, Massachusetts, United States of America; University of North Carolina School of Medicine, United States of America

## Abstract

The exonuclease1 (*Exo1*) gene is a key component of mismatch repair (MMR) by resecting the damaged strand, which is the only exonuclease involved in the human MMR system. The gene product is a member of the RAD2 nuclease family and functions in DNA replication, repair and recombination. However, whether *Exo1* is required to activate MMR-dependent DNA damage response (DDR) remains unknown, the conclusions of the *Exo1* polymorphisms on cancer susceptibility studies were not consistent. We carried out a meta-analysis of 7 case-control studies to clarify the association between the *Exo1* K589E polymorphism and cancer risk. Overall,a significant association of the *Exo1* K589E polymorphism with cancer risk in all genetic models (Lys vs Glu: OR = 1.51, 95%CI:1.39–1.99, *P*<0.01; Glu/Lys vs Glu/Glu: OR = 1.43, 95%CI:1.28–1.60, *P*<0.01; Lys/Lys vs Glu/Glu: OR = 2.45, 95%CI:1.90–3.17, *P*<0.01; Lys/Lys+Glu/Lys vs Glu/Glu: OR = 1.53, 95%CI:1.38–1.71, *P*<0.01; Glu/Glu vs Glu/Lys+Lys/Lys: OR =  2.27, 95%CI:1.79–2.89, *P*<0.01). In the stratified analysis by ethnicity, significantly increased risk was observed in Asian population (Lys vs Glu: OR = 1.53, 95%CI:1.39–1.69, *P*<0.01; Glu/Lys vs Glu/Glu: OR = 1.50, 95%CI:1.34–1.69, *P*<0.01; Lys/Lys vs Glu/Glu: OR = 2.48, 95%CI:1.84–3.34, *P*<0.01; Lys/Lys+Glu/Lys vs Glu/Glu: OR = 1.58, 95%CI:1.41–1.78, *P*<0.01; Glu/Glu vs Glu/Lys+Lys/Lys: OR = 2.18, 95%CI:1.62–2.93, *P*<0.01). Subgroup analysis based on smoking suggested *Exo1* K589E polymorphism conferred significant risk among smokers (Lys/Lys+Glu/Lys vs Glu/Glu: OR = 2.16, 95%CI:1.77–2.63, *P*<0.01), but not in non-smokers (Lys/Lys+Glu/Lys vs Glu/Glu: OR = 0.89, 95%CI:0.64–1.24, *P* = 0.50). In conclusion, *Exo1* K589E Lys allele may be used as a novel biomarker for cancer susceptibility, particularly in smokers.

## Introduction

Cancer is the leading cause of death in economically developed countries and the second leading cause of death in developing countries [1]. In the United States, one fourth deaths are due to cancer [2]. The burden of cancer is increasing in economically developing countries as a result of population aging and growth as well as, increasingly, an adoption of cancer-associated lifestyle choices including smoking. Primary prevention strategies aim to reduce incidence, the early detection as subclinical cancer cases are discovered, which increases the chance of a cure in early stage patients or prolongs their survival time. However, most cancers are difficult to detect at their early stage, new markers for identifying high-risk populations as well as novel strategies for early detection are urgently needed. Now, mechanism of carcinogenesis is poorly understood. It has been suggested that susceptibility genes combining with environmental factors may be important in the development of cancer [3], [4].

Individual variation in genetic backgrounds can in turn result in different consequences following the environmental exposure and may ultimately determine cancer risk. DNA repair genes form a complex network that protect the genome's integrity from endogenous and exogenous damage [5]. When DNA damage is not repaired and does not induce apoptotic elimination of the cell, DNA defects accumulate and are propagated through the cell progeny, and finally cancer may occur [6], [7]. Individual variations in DNA repair capacity due to the presence of polymorphisms in DNA repair-related genes may account for some cancer susceptibility in the general population [8], [9]. Genetic polymorphisms of DNA repair genes have been reported to determine susceptibility to several cancers [10]–[15].

The exonuclease1 (*Exo1*) gene, located at chromosome 1q42–43, contains one untranslated exon followed by 13 coding exons and encodes an 846 amino acid protein [16], [17]. The gene product is a member of the RAD2 nuclease family and functions in DNA replication, repair and recombination [18]. *Exo1* is a key component of mismatch repair (MMR) by resecting the damaged strand, however, whether Exo1 is required to MMR-dependent DNA damage response (DDR) remains unknown [19]. The conclusions of the *Exo1* polymorphisms on cancer susceptibility studies remain inconsistent, which is partially attributed to the heterogeneity of the cancer subtype, small sample size, and ethnicity of the patients.

A guanine (G)/adenine (A) common single nucleotide polymorphism (SNP) at first position of codon 589 in exon 13 of *Exo*1 (dbSNP ID: rs 1047840), resulting in the substitution of a glutamic acid (Glu, E) residue (GAG) by lysine (Lys, K) residue (AAG) (also designated *Exo1* K589E) in the exonic splicing enhancer (ESE), has been suggested to influence the products of *Exo1* mRNA. To further determine whether there is an association of the *Exo1* K589E with the risk for developing cancer, a comprehensive review and analysis of published data from different studies is needed.

In the present study, we have extensively reviewed literature and performed a meta-analysis based on all eligible case-control published data to evaluate the association between *Exo1* K589E polymorphisms and cancer susceptibility.

## Materials and Methods

### Identification of eligible studies

A comprehensive literature search was conducted using the PubMed, Springer, Elsevier, CNKI (Chinese), and Wanfang (Chinese) Digital Dissertations Databases for relevant articles published in English and Chinese up to December 2013 with key words ‘K589E/rs1047840’, ‘*Exo1* polymorphism’, and ‘cancer’. The full text of the candidate articles were examined carefully to determine whether they accorded with the inclusion criteria for the meta-analysis. The inclusion criteria were as follows: 1) about the *Exo1* K589E polymorphism and cancer risk, 2) from a case-control designed study, 3) sufficient published data for estimating an odds ratio (OR) with 95% confidence interval (CI), and 4) genotype frequencies available.

The studies, in which the genotype of controls for a certain polymorphism was not consistent with Hardy-Weinberg equilibrium (HWE) were excluded from the analysis of this polymorphism.

### Data extraction

Data were extracted independently by two investigators. For conflicting evaluations, an agreement was reached following discussion. If they could not reach a consensus, the third investigator was consulted to resolve the dispute, and a final decision was made by vote.

The following variables were extracted from each study if available: first author's name, publication year, cancer type, country of origin, ethnicity, study design, genotype distributions, and HWE of controls, respectively. Different ethnicity descents were categorized as Asian or Caucasian. Study design was stratified into hospital-based study and population-based study. If original genotype frequency data were unavailable in relevant articles, a request for additional data was sent to the corresponding author.

### Statistical analysis

The analyses were conducted in Review Manager 5.0. The risks (ORs) of cancer associated with *Exo1* K589E polymorphism were calculated directly from the data given in the eligible studies. OR corresponding to 95%CI was used to assess the strength of association between *Exo1* K589E polymorphism and cancer. The pooled ORs were performed for allelic comparison (Lys vs Glu), heterozygote comparison (Glu/Lys vs Glu/Glu) and homozygote comparison (Lys/Lys vs Glu/Glu), dominant model (Lys/Lys+Glu/Lys vs Glu/Glu), recessive model (Glu/Glu vs Glu/Lys + Lys/Lys), respectively. Furthermore, studies were stratified according to ethnicity (Asian, Caucasian) and smoking status.

We assessed the departure from the HWE for the control group in each study using Pearson's goodness-of-fit *χ^2^* test with 1 degree of freedom.

Heterogeneity in meta-analysis refers to the variation in study outcomes between different studies. Between-study heterogeneity was evaluated with a *χ^2^* based Q-test among the studies [20]. Heterogeneity was considered significant when *P<0.05*. In case of no significant heterogeneity, point estimates and 95%CI was estimated using the fixed effect model (Mantel-Haenszel), otherwise, random effects model (DerSimonian Laird) was employed [21], [22]. The significance of overall odds ratio (OR) was determined by the Z-test. If there were significant heterogeneity among included studies, the sources of heterogeneity would be explored using meta regression in Stata version 12.0 (http://www.stata.com).

To assess the stability of the results, one-way sensitivity analyses were performed to assess the stability of the results, in which a single study in the meta-analysis was deleted each time to reflect the influence of the individual data set to the pooled OR. The publication bias was diagnosed by using inverted funnel plots, Begg's test and the Egger's test by Stata 12.0.

Statistical tests performed in the present analysis were considered significant whenever the corresponding null-hypothesis probability was *P*<0.05.

## Results

### Study characteristics

**Figure 1:**
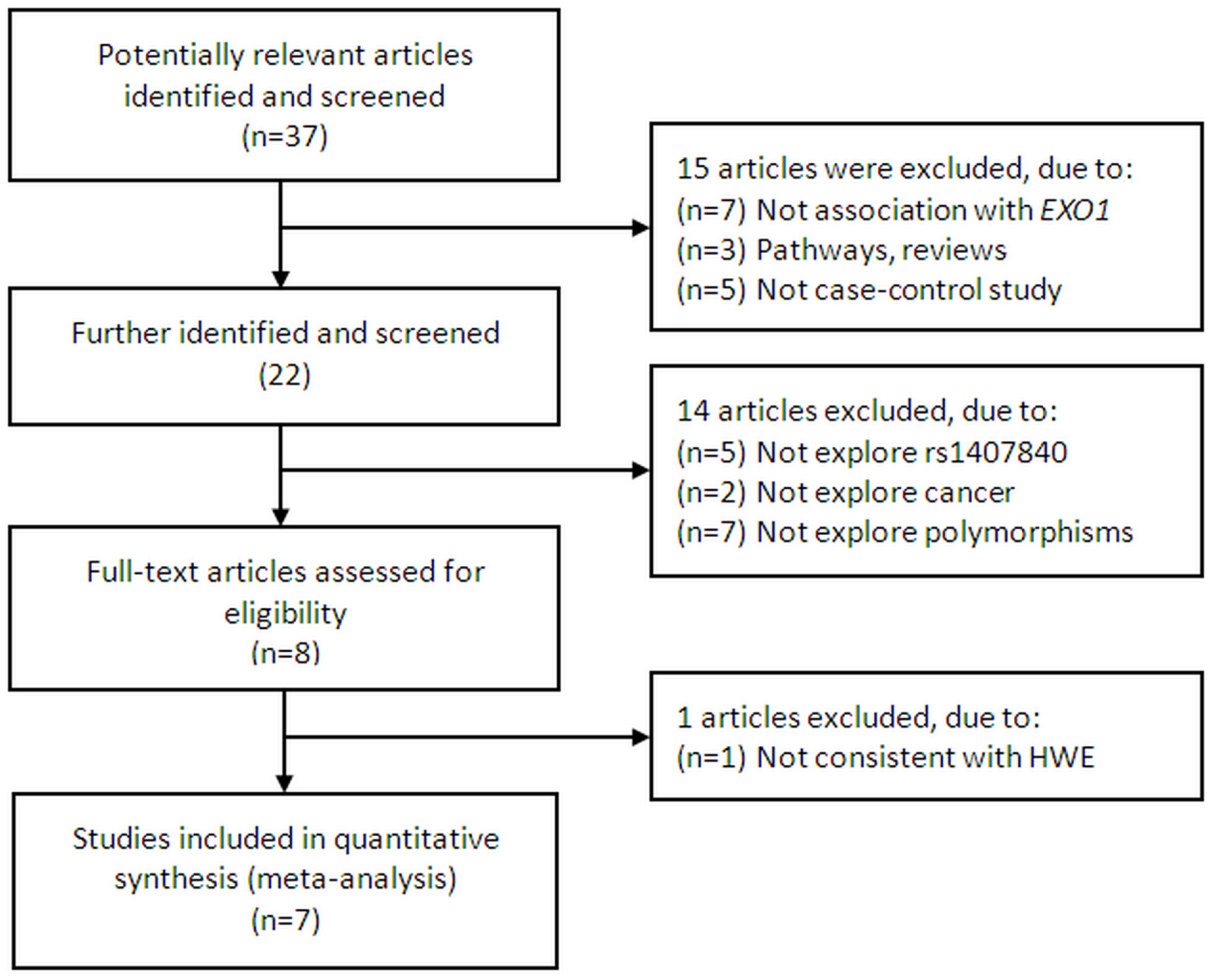
Flow chart of literature search and study selection.

A total of 8 publications met the inclusion criteria [23]–[30], as summarized in Table 1 (the study selection process was shown in Figure 1). In one article [24], genotype of controls for a certain polymorphism was not consistent with HWE, therefore, it was excluded from the analysis. Hence, a total of 7 studies including 2,951 cases and 3,101 controls were used in the meta-analysis. All studies were case-control studies, including 7 studies on 7 cancer types. There were 5 studies of Asian descendent and 2 of Caucasian descendent. A classic PCR-RFLP assay was used in 6 out of 7 studies. One study was randomly repeated a portion of samples as quality control while genotyping.

**Table 1:** Characteristics of studies included in the meta-analysis.

First author	Year	Ethnicity	Cancer type	Source of control	Genotyping	Matching criteria	Case/Control	Quality control^a^	HWE^b^ *P*-Value
Chang [23]	2008	Caucasian	Glioma	Population	Chip	Age; gender; ethnicity	112/110	NA	0.419
Jin [24]	2008	Asian	Lung cancer	Population	Chip	Age; gender	500/517	Y	0.030
Wang [25]	2009	Asian	Breast cancer	Population	PCR-RFLP	Age; gender	1272/1272	NA	0.926
Tsai [26]	2009	Asian	Oral cancer	Population	PCR-RFLP	Age; gender	680/680	NA	0.626
Hsu [27]	2009	Asian	Lung cancer	Population	PCR-RFLP	Age; gender	358/358	NA	0.864
Bau [28]	2009	Asian	Gastric Cancer	Population	PCR-RFLP	Age; gender	179/179	NA	0.940
Luo [29]	2012	Asian	Cervical Cancer	Population	PCR-RFLP	Ethnicity	126/278	NA	0.411
Bayram [30]	2012	Caucasian	Hepatocellular carcinoma	Population	PCR-RFLP	Age; gender; smoking; alcohol consumption	224/224	Y	0.089

a : Quality control: Quality control was conducted when sample of cases and controls was genotyped; NA: not available.

b : HWE: Hardy-Weinberg equilibrium in control.

### Quantitative synthesis

**Figure 2:**
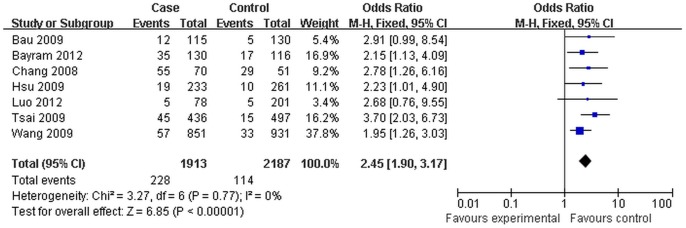
Forest plot of cancer risk associated with *Exo1* K589E for the homozygote comparison (Lys/Lys vs Glu/Glu). The squares and horizontal lines correspond to the study-specific OR and 95% CI. The area of the squares reflects the study specific weight. The diamond represents the pooled OR and 95% CI.

The main results of this meta-analysis and the heterogeneity test were shown in Table 2 (Figure 2). We firstly analyzed the association in the overall population. Then in order to obtain the exact consequence of the relationship between *Exo1* K589E polymorphism and cancer susceptibility, stratified analyses by ethnicity and smoking status were performed. When the Q-test of heterogeneity was not significant, we conducted analyses using the fixed effect models. The random effect models were conducted when we detected significant between-study heterogeneity.

**Table 2:** Main results of pooled ORs of the *Exo1* K589E polymorphisms on cancer risk in the meta-analysis.

Comparisons	Cases	Controls	Heterogeneity test			Summary OR *(95% CI)*	Hypothesis test		Studies
	n/N	n/N	*Q*	*P*	*I^2^*(%)		*Z*	*P*	
Lys vs Glu	1494/5899	1142/6202	6.79	0.34	12	1.51(1.39,1.99)	9.11	<0.01	7
Glu/Lys vs Glu/Glu	1038/2723	914/2987	7.98	0.24	25	1.43(1.28,1.60)	6.28	<0.01	7
Lys/Lys vs Glu/Glu	228/1913	114/2187	3.27	0.77	0	2.45(1.90,3.17)	6.85	<0.01	7
Glu/Lys+Lys/Lys vs Glu/Glu	1266/2951	1208/3101	6.49	0.37	8	1.53(1.38,1.71)	7.81	<0.01	7
Lys/Lys vs Glu/Glu+Glu/Lys	228/2951	114/3101	2.98	081	0	2.27(1.79,2.89)	6.67	<0.01	7

In the overall analysis, we found a significant association between *Exo1* K589E polymorphism and cancer risk in all genetic models (Lys vs Glu: OR = 1.51, 95%CI:1.39–1.99, *P*<0.01; Glu/Lys vs Glu/Glu: OR = 1.43, 95%CI:1.28–1.60, *P*<0.01; Lys/Lys vs Glu/Glu: OR = 2.45, 95%CI:1.90–3.17, *P*<0.01; Lys/Lys+Glu/Lys vs Glu/Glu: OR = 1.53, 95%CI:1.38–1.71, *P*<0.01; Glu/Glu vs Glu/Lys + Lys/Lys: OR =  2.27, 95%CI:1.79–2.89, *P*<0.01).

**Figure 3:**
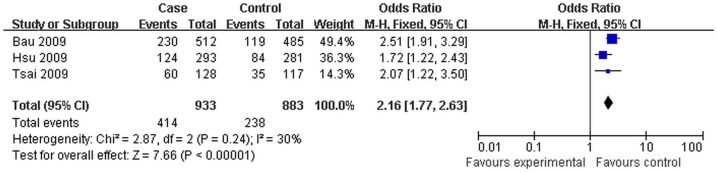
Forest plot of cancer risk associated with *Exo1* K589E for the dominant model (Lys/Lys+Glu/Lys vs Glu/Glu) in smokers. The squares and horizontal lines correspond to the study-specific OR and 95% CI. The area of the squares reflects the study specific weight. The diamond represents the pooled OR and 95% CI.

Further stratification analysis by ethnicity, the results showed that *Exo1* K589E polymorphism was significantly linked to cancer risk (table 3, figure 3). Overall, individuals carrying Lys allelic had a subtly increased cancer risk among Asian population (Lys vs Glu: OR = 1.53, 95%CI:1.39–1.69, *P*<0.01; Glu/Lys vs Glu/Glu: OR = 1.50, 95%CI:1.34–1.69, *P*<0.01; Lys/Lys vs Glu/Glu: OR = 2.48, 95%CI:1.84–3.34, *P*<0.01; Lys/Lys+Glu/Lys vs Glu/Glu: OR = 1.58, 95%CI:1.41–1.78, *P*<0.01; Glu/Glu vs Glu/Lys + Lys/Lys: OR = 2.18, 95%CI:1.62–2.93, *P*<0.01). In Caucasian population, *Exo1* K589E polymorphism was significantly associated with an increased risk in the allelic contrast, homozygote comparison and recessive model (Lys vs Glu: OR = 1.43, 95%CI:1.14–1.79, *P*<0.01; Lys/Lys vs Glu/Glu: OR = 2.37, 95%CI:1.44–3.97, *P*<0.01; Glu/Glu vs Glu/Lys + Lys/Lys: OR = 2.48, 95%CI:1.64–3.75, *P*<0.01).

**Table 3:** Stratified analyses of the *Exo1* K589E polymorphism on cancer risk.

Comparisons	Heterogeneity test			Summary OR *(95% CI)*	Hypothesis test		Studies
	*Q*	*P*	*I^2^(%)*		*Z*	*P*	
Ethnic							
*Asian*							
Lys vs Glu	3.72	0.45	0	1.53(1.39,1.69)	8.58	<0.01	5
Glu/Lys vs Glu/Glu	1.54	0.82	0	1.50(1.34,169)	6.76	<0.01	5
Lys/Lys vs Glu/Glu	3.01	0.56	0	2.48(1.84,3.34)	5.96	<0.01	5
Glu/Lys+Lys/Lys vs Glu/Glu	2.69	0.61	0	1.58(1.41,1.78)	7.93	<0.01	5
Lys/Lys vs Glu/Glu+Glu/Lys	2.51	0.64	0	2.18(1.62,2.93)	5.14	<0.01	5
*Caucasian*							
Lys vs Glu	2.74	0.10	64	1.43(1.14,1.79)	3.10	<0.01	2
Glu/Lys vs Glu/Glu	0.10	0.75	0	0.93(0.66,1.33)	0.38	0.70	2
Lys/Lys vs Glu/Glu	0.25	0.62	0	2.37(1.44,3.97)	3.37	<0.01	2
Glu/Lys+Lys/Lys vs Glu/Glu	0.98	0.32	0	1.17(0.84,1.63)	0.95	0.34	2
Lys/Lys vs Glu/Glu+Glu/Lys	0.18	0.67	0	2.48(1.64,3.75)	4.29	<0.01	2
Smoking status							
*smokers*							
Glu/Lys+Lys/Lys vs Glu/Glu	2.87	0.24	30	2.16(1.77,2.63)	7.66	<0.01	3
*non-smokers*							
Glu/Lys+Lys/Lys vs Glu/Glu	0.72	0.70	0	0.89(0.64,1.24)	0.68	0.50	3

Subgroup analysis was also stratified by smoking status. *Exo1* K589E polymorphism was significantly associated with an increased cancer risk in smokers (Lys/Lys+Glu/Lys vs Glu/Glu: OR = 2.16, 95%CI:1.77–2.63, *P*<0.01), but no association was observed in non-smokers (Lys/Lys+Glu/Lys vs Glu/Glu: OR = 0.89, 95%CI:0.64–1.24, *P* = 0.50).

### Evaluation of publication bias

**Figure 4:**
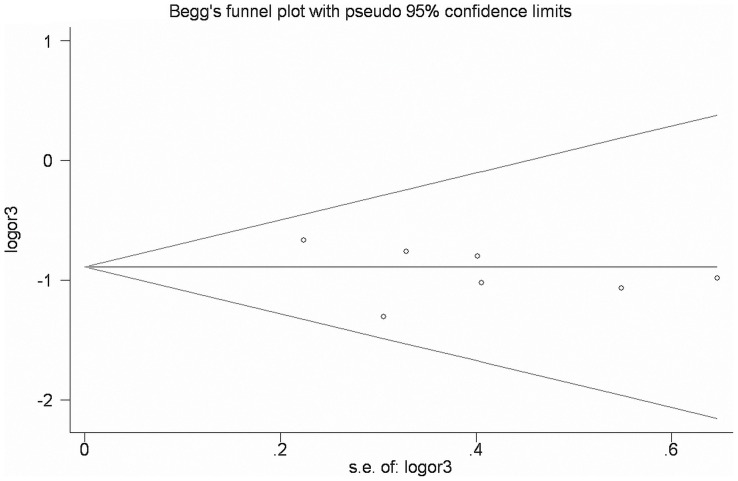
Funnel plot of *Exo1* K589E polymorphism and cancer risk for d homozygote comparison (Lys/Lys vs Glu/Glu).

Begg's funnel plot and Egger's test were performed to assess the publication bias of the currently available literature. The shape of the funnel plots did not reveal any evidence of obvious asymmetry in all comparison models (Figure 4). Then, the Egger's test was used to provide statistical evidence for funnel plot symmetry (Table 4).

**Table 4:** Publication bias of *Exo1* K589E for Egger's test.

Comparisons	*t*	*p*	*95% CI*
Lys vs Glu	−0.82	0.451	−3.906∼2.020
Glu/Lys vs Glu/Glu	−0.24	0.823	−3.145∼2.617
Lys/Lys vs Glu/Glu	−0.94	0.390	−3.137∼1.457
Glu/Lys+Lys/Lys vs Glu/Glu	−0.60	0.574	−2.907∼1.804
Lys/Lys vs Glu/Glu+Glu/Lys	−0.12	0.906	−2.796∼2.538

### Sensitivity analysis

A single study involved in the meta-analysis was deleted each time to reflect the influence of the individual data-set to the pooled ORs, and the corresponding pooled ORs were not materially altered, indicating that our results were statistically robust (data not shown).

## Discussion

*Exo1* is a member of the RAD2 family of nucleases and possesses 5′ to 3′ double-stranded DNA (dsDNA) exonuclease and 5′–flap endonuclease activities and functions in a number of important cellular pathways including DNA repair, replication, recombination, and telomere integrity [31]. Among the DNA repair system, *Exo1* is the only exonuclease involved in the human MMR system, one of the major roles is the MMR system which is responsible for correcting mismatches between bases and small insertion or deletion loops [32], [33]. Although many SNPs in *NQO1, CYP1A1, ERCC4, EXO1, MSH2, XRCC1* and *hOGG1* have been identified, only some of them have been extensively investigated in epidemiological studies [34], SNPs for which potential functional evidence in the development, progression and metastasis of cancer remains unknown, especially for *Exo1* gene.

In the present study, we were first analyzed the association of *Exo1* K589E of cancer from 7 studies. The pooled results revealed that *Exo1* K589E Lys allele was associated with an increased risk for developing cancer. Among Asian population, *Exo1* K589E polymorphism was significantly associated with an increased cancer risk in all genetic models but not in the Caucasian population, this suggested that a possible ethnic difference in the genetic background. Subgroup analysis was stratified by smoking status, *Exo1* K589E polymorphism was significantly associated with an increased cancer risk in smokers, but no significant association was observed in non-smokers. The reasonable explanation is cigarette smoking, a well-known origin of DNA damage, releases many DNA damage inducers to respiratory system and causes DNA damages to the cells. Therefore, people who have high-risk genetic variant, such as the Lys allele of K589E, and also smoking habits, the combined effect of genetic and environmental factors would synergistically increase their cancer susceptibilities.

Although meta-analysis is robust, our study still has some limitations. Firstly, lacking sufficient eligible studies limited our further stratified analysis on types of cancer. Secondly, for each selected case-control study, our results were based on unadjusted estimates, whereas a more precise analysis could be performed if individual data were available. Thirdly, lack of the original data of the reviewed studies limited our further evaluation of potential interactions, because the interactions between gene-to-gene and gene-to-environment may modulate cancer risk. Fourthly, although all eligible studies were summarized, the relatively small sample size of studies may lead to reduced statistical power when stratified according to the ethnicity and smoking status.

In summary, this meta-analysis suggested that the *Exo1* K589E polymorphism was significantly associated with increased risk of cancer, especially in smokers. However, further well-designed studies in large cohort of different ethnic origins and cancer types are needed before the application of *Exo1* K589E polymorphism as cancer biomarker in clinical settings and early cancer detection.

## Supporting Information

Checklist S1 **PRISMA Checklist.**
(DOC)
